# Improving Patient Outcomes: Effectively Training Healthcare Staff in Psychological Practice Skills: A Mixed Systematic Literature Review

**DOI:** 10.5964/ejop.v11i3.923

**Published:** 2015-08-20

**Authors:** Katherine Garzonis, Eryn Mann, Aleksandra Wyrzykowska, Pavlo Kanellakis

**Affiliations:** a Department of Clinical, Educational and Health Psychology, University College London, London, United Kingdom; b Saint Andrew's Healthcare, Northampton, United Kingdom; c Department of Psychology and Behavioural Sciences, Coventry University, Coventry, United Kingdom; d Kanellakis and Associates, London, United Kingdom; Aalborg University, Aalborg, Denmark

**Keywords:** psychology, skills, training, mental health professionals, professional development, communication, general practitioners, nurses, multidisciplinary team

## Abstract

Training is an important part of modern European healthcare services and is often cited as a way to improve care quality. To date, various training methods have been used to impart skills relevant to psychological practice in a variety of mental health professionals. However, patient outcomes are rarely used in evaluating the effectiveness of the different training methods used, making it difficult to assess true utility. In the present review, we consider methods of training that can effectively impact trainee and patient outcomes. To do so, PubMed, PsycNET, Scopus, CENTRAL and ERIC were searched for studies on training of healthcare staff in psychological practice approaches. In total, 24 studies were identified (16 quantitative and 8 qualitative). For the most part, group, individual, and web-based training was used. A variety of health professionals were trained in skills including ‘communication’, ‘diagnosis’, and ‘referral’ to name but a few. In the majority of studies staff skill level improved. These findings hold implications for the design, implementation, and evaluation of training for mental healthcare staff.

## Introduction

Every year almost 40% of people within the European Union (EU) suffer from some form of mental disorder (Wittchen et al., 2011). The recent increases in the number of diagnoses available (American Psychiatric Association, 2013) has contributed to more than 80 million additional people needing treatment in 2010 compared to 2005 (Wittchen et al., 2011), with as many as 200 million European citizens suffering from mental health problems today. However, only a third will access specialist mental health treatment (Wittchen et al., 2011), and the most common point of entry to services will be through doctors and other medical staff (Alonso et al., 2004; Gater et al., 2005). This creates a bigger need for psychologically-informed practice across healthcare systems, and places a burden on a range of healthcare professionals to develop these necessary skills.

Techniques for psychologically-informed practice are diverse in themselves, and recommendations to improve care have included developing communication skills (Travado, Reis, & Borras, 2014), cultural sensitivity (National Institute for Health and Clinical Excellence, 2011), and interpersonal interactions (de Haes & Tunissen, 2005) to name but a few. In addition to these abilities, the rapidly-expanding evidence base of diagnoses and their treatments creates a need to regularly update healthcare professionals' own psychological knowledge. ‘Education, training, and development’ are regularly acknowledged as the preferred way to enhance skill acquisition, and more frequently the only way to improve patient care in this context (Department of Health, 2013).

While training is an oft-cited concept, it is rarely recommended how it should occur. For example, the Francis (2013) report—based on an inquiry into the notorious Mid-Staffordshire Trust, an English National Health Service Centre with appallingly low standards of care—uses the term 'training' no fewer than 583 times. However, there is little discussion about the form this training should or could take. The challenges posed by the current financial climate mean there is a need now more than ever for efficacious learning, yet there is little consideration for what makes healthcare training effective. Undeniably, the end-goal of most professional development within mental health is to improve patient outcomes; it is therefore reasonable to assume effective training positively impacts patients. However, there are relatively few studies defining effective training of healthcare staff in this way, and fewer still that include considerations on the methods of training.

### The Present Review

More people than ever before are presenting with mental health difficulties to general healthcare services (Alonso et al., 2004; Gater et al., 2005; Wittchen et al., 2011). Mental health has become a matter for all health professionals (Department of Health, 2013) and a cross-disciplinary approach can produce “significant added value” (European Parliament, 2009). This widening scope of mental health care-related roles, combined with financially-stretched healthcare systems, means a multidisciplinary approach to psychological skills development is not only important, but timely. Thus, we will examine the training provided to a range of staff who work with patients in mental health settings, including, but not limited to, psychologists, nurses, and physicians. These staff will be collectively referred to as Mental Health Professionals (MHPs) from here.

While there are many ways to define concepts as variable as 'skills' and 'training', this review will focus on training as “the systematic development of attitude, knowledge, skill, behaviour patterns required by an individual in order to perform adequately a given task or job” (Department of Employment, 1971). Our understanding of 'skill' follows the Cambridge Dictionary's (“Skill,” 2014): “an ability to do an activity or job well”, where this refers to a specific pattern of behaviour. Thus 'psychological skills training' can be understood as a systematic effort to create specific behaviour patterns relevant to good psychological practice.

While training frequently forms a significant part of recommendations to improve patient care (e.g. Webb, 2011; World Health Organization, Global Health Workforce Alliance, 2008), what constitutes the most *effective* types of training is frequently overlooked. In order to bridge this gap, the aim of the present review is to investigate ‘*what method of training is most effective in teaching psychological practice skills to MHPs?*'

To understand the efficacy of training, it is necessary to consider both patient and staff outcomes–especially as training may be enjoyable and change behaviour in trainees, but still have no benefit to patients (Hinrichs, 1976). To the authors' knowledge, at the time of writing this type of analysis of psychological practice skills training among MHPs has not yet been undertaken, but, as described previously, is sorely needed.

## Method

### Search Strategy

Searches were made of databases PsycNET, Scopus, PubMed, CENTRAL (The Cochrane Library), and ERIC (Institute of Education Studies) for publications between 2009 and 2014. The same search terms and search structures (e.g. nested searches) were used in each database. Where possible, filters appropriate to pre-define inclusion and exclusion criteria were included. Identical search criteria were used to identify both qualitative and quantitative data from studies. By not specifying the research methodology, we minimised the number of appropriate studies missed through inadequate filters (Gorecki et al., 2010).

Added to these results were any relevant documents identified from the resultant studies' citations, which were subject to the same inclusion and exclusion criteria. Study quality was evaluated using the Critical Appraisal Skill Program Qualitative Research Checklist (CASP, 2013) for qualitative studies, and the Mixed Methods Appraisal Tool (Pluye & Hong, 2014) for all other study designs. All reviewers carried out the same search on one of the databases in order to check consistency between searches and quality ratings.

### Search Terms

The search terms used in the current review are presented in Table 1, below. Nested searches are indicated by terms within brackets. 'Muscle' was used as an exclusion term to reduce the number of spurious results related to muscle training.

**Table 1:** Search Terms and Their Operators for Locating Studies

Contains at least one	Must contain	Does not contain
"Continuing professional" **OR** development **OR** CPD **OR** education **OR** training **OR** learning **OR** supervision **OR** skill **OR** skills	**AND** (nurse OR nurses OR healthcare OR “health care” OR psychologist OR psychologists OR doctor OR doctors) **AND** (patient OR patients OR client OR clients OR “service user” OR “service users”)	**NOT** muscle

Terms related to skills, training and their synonyms were identified from prior reading of related literature (Higgins & Green, 2011) and have been cross-referenced with Index Terms on PsycNET to generate a greater number of relevant results. The search process was evaluated through consultation with senior researchers involved in the project. Searches were also checked to see whether pre-identified papers of relevance were picked up by the strategy. Multiple trials of different search terms and strategies were used in order to get the most appropriate results. These trials suggested using broader search terms over more specific ones in order to pick up the greatest number of relevant studies.

In deciding terms for MHPs, we have been selective in adding common key words likely to target the largest proportion of workers. The sheer number of professionals involved in mental health alone has necessarily meant we have not included some specific MHP terms in order to keep search results manageable. The charity Mind for example lists 37 different professions in mental health (Challis, 2013), and therefore it has not been possible to include them all in this search.

### Criteria for Selecting Studies

Table 2 lists the inclusion and exclusion criteria for studies in this review. These were designed to select high-quality studies with both staff- and patient-outcome data.

**Table 2:** Inclusion and Exclusion Criteria

Inclusion	Exclusion
Staff outcomes included skills relevant to psychological practice (e.g. communication, formulation) Staff were adults Peer-reviewed Qualitative, mixed, or quantitative studies (e.g. reviews, trials, or RCTs) If filtering was available, studies were listed under an educational/training heading Patient outcomes Articles in English, Polish, Greek, Punjabi, or Spanish^a^	Quantitative studies without comparison data (outcome pre- and/or post-intervention, or control group) Insufficient description to replicate programme delivery (e.g. length of intervention, time between sessions, etc.) Full text cannot be found Papers written in a language not understood by the reviewers Intervention was not delivered to healthcare staff Studies on non-human subjects Developmental studies (e.g. childhood or ageing studies) Skills irrelevant to psychological practice in an adult mental healthcare setting were taught

^a^Languages understood by reviewers.

We have used staff- and patient-level outcomes to indicate effectiveness of interventions. A patient outcome was defined as: “a likely consequence of training that is meant to impact patient mental health”, where that impact is referenced in the Department of Health's current policies on improving mental health outcomes (Department of Health, 2011a, 2011b, 2014; Public Health England, 2013). This understanding was based on a general definition of 'outcome' (“Outcome,” 2015) with the addition of a specific focus on mental health as informed by government policy. Staff outcomes were defined as: “a likely consequence of training meant to impact staff skill(s)”, following our earlier definition of skills.

## Results

The search strategy identified over two thousand papers across the five search engines (Table 3). To be included in the analysis a study needed to describe a training process, report a trainee outcome linked to training effectiveness, and include a patient outcome that could potentially be connected to effects of training. Most studies were excluded as they did not to describe the training of MHPs, and others were screened out at later stages because they did not report patient outcomes or detailed descriptions of training procedures. After application of all the inclusion/exclusion criteria, the number of studies analysed was 29. Five further studies were removed afterwards as it was decided 'standardised patients', played by actors, could not be used to indicate patient outcomes. In total, 24 studies were eligible for the final analysis, including eight qualitative studies.

**Table 3:** Search Results

Stage	Nº studies examined	Nº studies excluded	Reason for exclusion
Articles obtained from search terms	2421	967	Irrelevant topic
Abstracts retrieved for evaluation	454	157	Did not meet criteria
Articles attempted to find full text for	297	83	Full text not accessible
Full text obtained for detailed evaluation	214	185	Did not meet criteria
Articles considered for analysis	29	5	Used standardised patients
Articles finally included	24		

### Review of Studies

For clarity of description, the selected studies have been grouped into quantitative or qualitative methodologies, followed by a consolidative summary. Following Cochrane guidelines (Higgins & Green, 2011), quantitative papers were reviewed using a narrative approach, while qualitative studies were examined using a form of thematic synthesis (Thomas & Harden, 2008). Finally, the conclusions of these analyses were combined into a united synthesis, following a method of Bayesian conversion (The Joanna Briggs Institute, 2014).

Most studies had a CASP or MMAT quality rating of 50% or higher (see Table 4). Qualitative studies on average scored more highly than quantitative methodologies, which may be because few randomised control trials adequately described their blinding process.

**Table 4:** Summary of Included Studies

Authors	Year	Design	Training Type	Trainees	Quality Score^a^
Aiarzaguena et al.	2009	Qualitative	Group	GPs	6/7
Amerson	2012	Qualitative	Individual + group	Nurses	7/7
Bakker et al.	2010	RCT	Group	GPs	50%
Bregnhøj, Thirstrup, Kristensen, Bjerrum, & Sonne	2009	RCT	Individual + group	GPs	75%
Collin, Valleala, Herranen, & Paloniemi	2012	Qualitative	Individual + group	Emergency staff, including doctors and nurses	6/7
Feder et al.	2011	RCT	Group	Multidisciplinary clinical teams	100%
de Feijter, de Grave, Dornan, Koopmans, & Scherpbier	2011	Qualitative	Individual	Medical students	7/7
Fossli Jensen et al.	2011	RCT	Group	Hospital doctors	75%
Gongora-Ortega, Segovia-Bernal, Valdivia-Martinez, Galaviz-deAnda, & Prado-Aguilar	2012	RCT	Individual + group	GPs	75%
Guldin, Vedsted, Jensen, Olesen, & Zachariae	2013	RCT	Individual	GPs	75%
Jansink et al.	2013	RCT	Individual + group	Nurses	25%
Karlin et al.	2012	Non-controlled	Group	Cognitive behavioural therapists	100%
Lester, Birchwood, Freemantle, Michail, & Tait	2009	RCT	Group	GPs	50%
Mackey & Dodd	2011	Non-controlled	Individual + group	Managers of learning disability services	100%
McLoughlin	2010	Qualitative	Group	Child and Adolescent Mental Health Services psychodynamic therapists	5/7
Monrouxe & Rees	2012	Qualitative	Individual	Medical students	6/7
Nilsen	2011	Qualitative	Web-based video	GPs and hospital specialists	6/7
Penner et al.	2013	RCT	Individual	Physicians	25%
Shirazi et al.	2013	RCT	Group	GPs	75%
Simons et al.	2010	Non-controlled	Individual + group	Cognitive behavioural therapists	100%
Suldo, Friedrich, & Michalowski	2010	Qualitative	Group + presentations	School psychologists	6/7
Tarn, Paterniti, Orosz, Tseng, & Wenger	2013	RCT	Group	Physicians	100%
van Weert, Jansen, Spreeuwenberg, van Dulmen, & Bensing	2011	RCT	Individual	Nurses	100%
White & Winstanley	2010	RCT	Individual + group	Mental health nurses	50%

^a^Scores out of 7 reflect CASP scores; percentage scores represent MMAT criteria.

A full summary of the review findings from both qualitative, quantitative, and synthesised analyses can be found in the Appendix.

### Quantitative Studies

#### Sample

Sixteen studies described training experiences with MHPs, who were most commonly general practitioners (GPs) or nurses. There was great heterogeneity in outcomes and training methods, which has prevented a meta-analysis. Results are therefore presented as a narrative, and conclusions should be treated tentatively as generalisations to the population cannot be assumed.

The training received by MHPs can be classed into ‘group’, ‘individual’, or ‘web-based’ categories. While there were some differences in how these types of training were applied across studies, in general group training involved a number of MHPs receiving the same training, ranging in length from 1-hour to 4-days. This type of training was often based in interactive approaches, including discussions, debates, and role-plays. Individual training typically involved trainees independently acquiring new skills. This was achieved by a variety of learning resources, including pamphlets, information sheets, or feedback. Finally, web-based training typically taught new skills through an online medium. Web-based training most often involved video-conferencing as a means to give feedback and tutorials to MHPs about their performance.

#### Group Training

Group training was used to teach communication skills to nurses (Jansink et al., 2013), GPs (Tarn et al., 2013), hospital doctors (Fossli Jensen et al., 2011), and learning disability staff (Mackey & Dodd, 2011). Across all studies, communication improved post intervention, although there is evidence to suggest this is not maintained over time (Jansink et al., 2013) or only to a small degree (Fossli Jensen et al., 2011). These group training sessions were interactive, as opposed to a traditional lecture format, including role-plays, discussions, etc.

Group training was also used to enhance GPs' diagnostic skills (Bakker et al., 2010; Feder et al., 2011; Shirazi et al., 2013), and again improvements were shown post intervention. Interestingly, interactive group training was superior to education alone when a direct comparison was made (Shirazi et al., 2013), and an exploratory analysis of these studies' effect sizes suggests such interactivity may more effectively teach diagnostic skill than group lectures alone.

Finally, improvements in skill were noted when group training was used to train mental health nurses in supervision skills (White & Winstanley, 2010) GPs in prescription skills (Bregnhøj et al., 2009). As such, it seems that group training may be efficacious in training MHPs in communication, diagnostic, supervision and prescription related skills.

Not all group training improved MHP skill level. When used to train referral skills in GPs (Feder et al., 2011; Lester et al., 2009), only one study detected an increase in ability (Feder et al., 2011). In the other study, the authors noted that educating GPs alone may be insufficient to make a difference in their skill level. They argue that as primary care is just one part of the care system; a whole-systems approach is required (Lester et al., 2009). Also, this study did not explicitly use interactive groups as a teaching method, unlike the study that found a significant difference.

Three other studies that found mixed results with non-interactive groups looked at training therapists (Karlin et al., 2012; Simons et al., 2010) and GPs (Gongora-Ortega et al., 2012) in clinical competency skills. Improvements were noted in only two of the three post-intervention, although it is worth noting that these studies sustained educational support at least 6 months longer than Gongora-Ortega and colleagues (2012). The latter's authors suggested the statistically non-significant result was a function of the small sample size, which may have led to insufficient power to detect a significant difference in pre- versus post-intervention scores (Gongora-Ortega et al., 2012).

The following comments on patient outcomes must be treated cautiously. It is not within the scope of this study to draw conclusions on the effectiveness of particular interventions on particular outcomes, as the number of studies is too small and too varied to facilitate strong conclusions. Given this, in general, patients seen by MHPs with skills obtained through group training were more likely to be referred for treatment (Bakker et al., 2010; Feder et al., 2011; Lester et al., 2009; Shirazi et al., 2013)—which can be counted as a patient outcome under the “No Health Without Mental Health” policy, relating to access (Department of Health, 2011a)—and see improvements in their depression and/or anxiety scores with treatment (Karlin et al., 2012; Simons et al., 2010).

Only one study, by Gonogora-Ortega and colleagues, of the four involving group training was able to improve patient satisfaction or trust in their MHP (Fossli Jensen et al., 2011; Gongora-Ortega et al., 2012; Tarn et al., 2013; White & Winstanley, 2010). However, Fossli Jensen et al. (2011) as well as White and Winstanley (2010) argue in some cases this may have been because satisfaction was already high, producing a ceiling effect.

Although about one third of the above studies mixed group training with additional individual work, there is not enough evidence to suggest this has a specific impact on either trainee or patient outcomes. Anecdotally, there is evidence to suggest interactive groups may enhance the acquisition of some skills in trainees. However, no such tendency was found regarding patient outcomes.

#### Individual Training

Studies using individual training showed less methodological variation than the group interventions. While group training would often involve a mixture of role-plays, discussions, presentations, etc.—with and without additional individual learning—the two studies using purely individual training tended to focus on only one or two approaches. This is to be expected, particularly as the above-mentioned activities require more than one person; individual learning is naturally limited here.

Penner et al. (2013) used written information as an aide-mémoire to convey a sense of 'being part of a team', while Guldin and colleagues (2013) supplemented their leaflets on recognising complicated grief with patient risk assessments for the same. Neither study was able to sustain their trainee effects at follow-up, suggesting written material alone is not sufficient to produce significant change. However, patients' trust in their doctor and adherence to medical advice was maintained at 16 weeks follow-up (Penner et al., 2013), as were small reductions approaching significance in depression and grief scores after one year (Guldin et al., 2013). This may indicate the interesting possibility that, for certain skills, MHP learning does not need to be sustained in order to produce long-standing patient changes. This may be more likely under conditions where the MHP has a time-limited role in the early stages of the care pathway. For example, a GP trained to give the most appropriate referral at the first signs of mental distress can greatly impact future care and course of the illness (Ross & Hardy, 1999). Further research in this area would be interesting, especially if such change is found more generally, such as by eliminating other potential factors like patient group.

#### Web-Enabled Training

As opposed to the above-mentioned group and individual training methods, in which the majority of learning aids are based in physical reality, a study by van Weert et al.'s (2011) used digital methods to create and host learning material. They used web-enabled video to record and upload individual nurses' consultations with cancer patients, which the nurses could watch at home or in the hospital. Individual feedback on communication skills was provided, and supplemented with a reflection task that encouraged the trainees to review their own material. This was linked to a small, statistically significant improvement in nurses' communication skills and patients' recall of consultation information compared to controls. However, an exploratory comparison with effect sizes from other studies targeting communication skills would suggest web-enabled training may be less effective at teaching this particular skill.

### Qualitative Studies

Data was extracted using a meta-ethnographic approach, wherein summary terms and concepts were identified from the authors' original findings and listed (Campbell et al., 2003). These were then used as codes and organised into themes and sub-themes, following a method of thematic synthesis (Thomas & Harden, 2008). These themes were re-examined and regrouped (as appropriate) in order to best represent the meanings in the code groups in the context of training methodology. Three other review authors appraised these to agree a final version, resulting in nine themes related to effective training.

#### Sample

A total of eight qualitative studies were selected for inclusion in the present review. Similar to the quantitative studies above, training involved: ‘group’ (Aiarzaguena et al., 2009; McLoughlin, 2010; Nilsen, 2011), ‘individual’ (Amerson, 2012; Collin et al., 2012; Monrouxe & Rees, 2012), and ‘web-based’ (videoconferencing; Nilsen, 2011) methods. In addition, training by means of ‘peer presentations’ was also identified (Suldo, Friedrich, & Michalowski, 2010).

#### Theme 1: Translation of Policy Into Practice

The challenges of applying what has been learnt within an educational environment to the healthcare setting was made clear in the research by Monrouxe and Rees (2012). Here, medical students reported that qualified staff members would often demonstrate actions inconsistent with their teaching, resulting in students experiencing contradictory formal and informal learning experiences. This reflects the quantitative work of Lester and colleagues (2009) and also White and Winstanley (2010) who both noted how the overall organisation can be a significant force, even barrier, to the implementation of skills learnt in training.

Monrouxe and Rees (2012) highlight peer support within the workplace as a potential coping strategy for staff in this situation. Their research shows this can help students overcome the negative emotional outcome caused by contradictions between teaching and practice. Mentoring, or other sources of peer support, may provide individuals with an awareness of professional issues within the learning environment, and help empower them toward articulation of their experience and positive cultural change. Moreover, Monrouxe and Rees (2012) suggest this ensures a respectful, professional service for all patients by encouraging awareness of such ethical issues.

#### Theme 2: The Training Environment

Although it can be difficult to translate learning into practice, the training environment can be utilised to improve generalisability of skills. De Feijter et al. (2011) argue the natural complexity of a healthcare environment means learning within the workplace can be more effective than a relatively simple classroom setting. In their study students were exposed to a variety of *in vivo* experiences, such as collaboration and time constraints, and therefore were presented with 'a realistic account' of what clinical practice entails. This type of training was described in relation to the Activity Theory (Engeström, 2006), where interactions between practitioners, customers (patients), and systems can be reflectively designed into the environment. This demonstrates the importance of insight into both becoming a healthcare professional and taking care of patients without doing harm, which can be used for better integrated and expansive design of training systems.

De Feijter et al. (2011) also identified the relationship with the supervisor as a key moderating factor in students' experience of the environment. As knowledge and trust in the trainee's abilities increased, they would gradually be given more responsibility by their supervisor. This exposed them to new and more complex interactions within the healthcare environment, allowing more advanced skill development.

#### Theme 3: Professional Culture in Congruent Care

In addition to where training is provided, the type of training provided should also be considered. Monrouxe and Rees (2012) highlight a 'clash of cultures' that can occur in training. They point out that trainers may be educated in alternative models of care, often those that erode the ‘patient-centred approach’. In turn, this can create a negative emotional state for the students, who, although they felt a strong identity with their teaching team, were morally distressed by providing healthcare that was insensitive to patients’ needs (Monrouxe & Rees, 2012). The findings suggest educationalists should be up to date with policies and expectations of the clinical setting. Doing so ensures culturally relevant practice, meaning patients receive appropriate care that is consistent with their expectations of the NHS (Monrouxe & Rees, 2012).

#### Theme 4: Understanding Individual Patients' Needs

The needs of patients should also be a primary concern when training is provided. For example, Aiarzaguena and colleagues (2009) found that targeting interventions at the consultation stage helped GPs feel closer to “difficult” patient groups, and also promoted better understanding of patient needs. Nilsen (2011) found that training within a multi-disciplinary team (MDT) promoted faster and more effective diagnosis. This meant that all professionals were able to contribute their expertise in considering different patient needs, therefore optimally delivering high-quality care. It was also found that specialists benefited from the collaboration, as it allowed for continuity both within the team and for the patient.

#### Theme 5: Patient Safety

In terms of the patient experience, Monrouxe and Rees (2012) also commented on how patients’ dignity and safety can be compromised during student training. They note this compromise was partly due to the amount of unsupervised student involvement within patient care. In their study, it was accepted that students were bound to make mistakes, yet they were also asked to practice beyond their level of competency. It was also found that consent was often not gained from patients for treatment by students, which pressurised students to take full responsibility for the care provided.

Finding the balance between learning through experience and reducing the potential harm for patients in healthcare settings should be an important concern, yet Monrouxe and Rees (2012) found that the standard of patient care is often compromised within medical training. De Feijter et al. (2011) suggest that approachable supervisors may be one means to support students to provide a high standard patient care and reduce the number of risks taken during training.

#### Themes 6-9: The Four Cs—Clarification, Co-ordination, Communication and Collaboration

Four common factors found consistently throughout all of the qualitative literature were: **clarification**, **co-ordination**, **communication** and **collaboration**. Collin and colleagues (2012) describe the importance of each of these factors when learning in emergency work.

Clear guidelines used within training for GPs were found to increase the application of skills to a variety of patients’ needs using a structured intervention technique (Aiarzaguena et al., 2009). This demonstrated the importance of clarification for skill-sets.

Co-ordination between the multidisciplinary team meant that learning could be on-going within the working environment; for example, when reviewing the patients’ needs on a daily basis using video-conferencing (Nilsen, 2011).

Fast and effective communication was a central feature of the qualitative research on learning within the workplace. Communication increased the speed and efficiency of patient diagnosis and planning of care (Collin et al., 2012).

Finally, when applied to a context of constant change, collaboration between the qualified member of staff, student and patient reduced the discrepancies within the healthcare training environment and maintained that the patients' needs were the priority of the service provided (Collin et al., 2012).

#### Structured and Unstructured Training

Delivery of training that did not follow a predetermined structure commonly took the form of learning within the 'here and now'; getting involved with all processes of patient care, often in collaboration with a senior member of staff (de Feijter et al., 2011). It appeared less common that training was provided within a pre-planned, structured manner for students.

Aiarzaguena et al. (2009) used a sample of GPs to test the use of a structured tool and a re-attribution technique for diagnosing patients with medically unexplained symptoms. It was found that by providing staff with training that used clear guidelines, their understanding increased and they were able to apply the framework taught to patients with psychological symptoms. The importance of relevant and regular training to improve insight for more challenging symptoms was highlighted by GP’s that completed the intervention.

#### Individual Learning

Individual learning was found to increase self-efficacy in cognitive, practical and affective learning dimensions, when students engaged in an international service-learning project for nursing (Amerson, 2012). Here, individual study, e.g. through reading assignments, improved cognitive understanding of cultural differences, which was complemented with the individual's experience of working in another country; “seeing makes it real” (Amerson, 2012, p. 12). These cultural insights translated into better communication skills, and thereby greater practical understanding of patient requirements. This improved patient outcomes as graduates were able to recognise culturally different responses to pain and were able to care for each individual’s specific needs more accurately.

Amerson (2012) also found that learning independently during international trips was effective training for allowing the learner to develop ‘a desire and passion to fulfil their job role to the best of their ability’. It was concluded in this study that exposing MHPs to a multitude of variables, such as: the treatment process, the form of treatment and the challenges of patient care—including exposure to inconsistencies within the practice—increased their competency and optimised learning interactions.

Collin and colleagues (2012) also reported on the use of individual learning involving all staff members working within an emergency unit for acute care. Errors occurring in the treatment chain were connected to inconsistent practice when working individually within a multi-professional collaboration process for patient care. For example, single staff roles overlapped without them even knowing about each other. However, within the study this was interpreted as learning through experience when mishaps were highlighted to employees, enabling them to learn from others’ experiences and mistakes. Individual-level learning was found to be common within the emergency work setting for patient care, as healthcare roles were represented as single, separate processes within the treatment chain for emergency practice.

#### Group Learning

As in the quantitative studies, training within a group was found to be the most common form of learning for MHPs, used with both qualified clinicians (Aiarzaguena et al., 2009; McLoughlin, 2010) and students (Amerson, 2012; de Feijter et al., 2011; Monrouxe & Rees, 2012).

Collaboration lead to higher quality of care for patients by improving the communication between multi-disciplinary teams, allowing for MHP’s to have better knowledge of patients.

An important feature of group learning was collaboration (Suldo, Friedrich, & Michalowski, 2010), which was linked to improvement in communication skills (Collin et al., 2012). This was most pronounced when working within a group at an early stage of contact with the patient, leading to a reduction in discrepancies when delivering practical instructions for patient care (Collin et al., 2012). Inter-professional collaboration facilitated learning as a natural part of care practices, whereby being a member of a multi-disciplinary team allowed for on-going learning within the workplace (Nilsen, 2011).

#### Video-Conferencing

Videoconferencing between general practitioners and hospitals in Norway was found to be most successful for efficient, collaborative work, with the ability to successfully adapt to the patient’s condition as it changed over time (Nilsen, 2011). By using videoconferencing as a communicative tool, healthcare professionals were able to exchange information in order to overcome any knowledge gaps relating to patients’ needs. Within this study, the regular use of videoconferencing meant a continuous follow-up of the patient’s progress, and allowing staff members to mutually share their expertise. Nilsen (2011) described this as separate activity systems coming together; the patient acts as a shared object uniting the healthcare professionals, providing a common activity and allowing for multi-disciplinary learning.

The selected patient case used to describe this training technique was discussed by the team over several days, allowing for a repertoire of knowledge to be developed. This improved the outcome for the patient, as their complex needs could be discussed as time unfolded allowing for continuity and prompt decision making with their treatment.

#### Peer Presentations

When given by MHPs, teaching through colleague presentations enhanced skill-sets of individuals within the team who did not specialise in mental health (Suldo, Friedrich, & Michalowski, 2010). It was found that solutions were generated before problems occurred, acting as a strong preventative method for risks. This would also improve the service-user access by providing interventions in more frequently used locations, such as at schools and within the workplace.

This finding was consistent with McLoughlin (2010), who found that when staff came together, it was possible to be constructive and creative, rather than work in a reactive way. In doing so, staff were able to listen to each other and take the time required to work through a complex situation (McLoughlin, 2010, p. 238).

### Synthesis

The data thus far has been presented as two separate analyses of quantitative and qualitative information. To unite these into a single summary, a synthesis is required. This has been achieved using a Bayesian-style conversion of quantitative-into-qualitative information, based on the method suggested by The Joanna Briggs Institute (2014).

First, the results of the quantitative analysis were written as descriptive—i.e. qualitative—statements. Second, these statements were compared to the conclusions of the qualitative analysis for topical similarity. Where similarity was identified, the original texts were referred to to determine the appropriate context of the statements (The Joanna Briggs Institute, 2014). Finally, a single statement reflective of both analyses was produced. These have been phrased in such a way as to give indications for practice, based on the available evidence.

#### Effectively Training in Psychological Practice Skills

The points below summarise the main findings of the synthesised material from the qualitative and quantitative studies. They have been divided into different methods of training, which were most amenable to a combined analysis.

1. Individual Training:
  - Individual training offers fewer opportunities for feedback, increasing the chance of inconsistencies in practice.
  - It is therefore more effective when combined with feedback from team members in related disciplines, particularly in MDTs.
  - Professional change from this type of training alone is unlikely to be permanent with a short period of study, but it can foster a passion for work that may motivate future efforts.
  - This motivation can contribute to improving patient outcomes that are based on single professional contacts early in the care pathway, such as trust in a doctor after one consultation.
  - As such, this training can be more efficient where short-term skill improvement is wanted, and can be expressed as written material.
2. Group Training:
  - Interacting within a group naturally, through exchanges, aids the development of communication skills and fosters learning, but requires an initial ability to collaborate effectively.
  - It is therefore most effective when a range of disciplines are represented, such as in MDTs, as this brings the biggest pool of relevant knowledge.
  - Outcomes that are affected by the availability of professional knowledge, such as referrals, diagnoses, care pathways, and (as a result) mental health symptoms, are thereby more effectively taught by such groups.
  - By the same token, group training has relatively little effect on outcomes like patient satisfaction, which do not depend as heavily on breadth of knowledge.
  - As more information and feedback is shared, practice becomes more consistent and knowledge-based skills improve with time.
3. Web-enabled Video Training:
  - As with group training, web-enabled video allows professionals to share knowledge and improve communication skill-related outcomes through interaction.
  - However, digitally-facilitated interaction requires several different communication practices specific to that medium, so is therefore not as effective as physically-present groups in enhancing face-to-face interaction abilities.
  - This method of training may then be more effective in training groups of remote individuals in communication- and knowledge-based skills.

## Discussion

Group, individual, and web-based training were used across quantitative and qualitative studies. Peer presentation was also used to train MHPs in the qualitative studies. Overall, each type of training positively impacted skills among MHPs. As such, the choice of training mode may be influenced by other factors such as resource availability, and the availability of trainers and trainees. Individual trusts may also have pragmatic or financial preferences for the type of training used among MHPs.

Some methods of training leant themselves more towards training particular abilities, especially communication skills, which appeared to be enhanced by group interactions with peers. This was related to a positive impact on patient outcomes that were dependent on MHP's ability to use and communicate knowledge, such as appropriate referrals and improvement in mental health symptoms. Other patient outcomes, such as satisfaction, proved more elusive, suggesting the selection of a training method should be considered in light of both the skill to be taught and the patient change this is hoped to impact.

### Implications for Clinical Practice

The studies reviewed suggest most methods of training are effective at improving skills in mental health professions, although this effect can be enhanced when type of skill and the desired patient outcome are taken into consideration. In general, it is suggested communication skills are more likely to improve with interactive, face-to-face groups, and this is more likely to improve patient outcomes that can be linked to availability of professional knowledge. Membership of an MDT is a reoccurring example of how this can be achieved even in an unstructured training environment, given appropriate collaboration.

Individual learning can be a useful way of making short-term skill changes, but is unlikely to create lasting change in trainees without significant time investment. Interestingly, the research suggests such change may not be necessary to positively affect patients when made early on in the care pathway. This further highlights the need for including patient outcomes when evaluating the effectiveness of learning interventions. The potential limitations of individual training should be considered in relation to the increase in 'e-learning' in many NHS trusts (e.g. Gray, Plaice, & Hadley, 2009), which is a short-term experience and generally a solitary activity.

Lastly, web-enabled video learning can be effective in teaching communication-based outcomes to remote participants when part of a group or as reflection, although this may not be as effective as groups occupying the same physical space.

### Research Implications

This review has contributed to the literature by examining what training can be effective at improving both patient and trainee outcomes in a wide range of studies. The large attrition rate from the initial literature search reflects the fact that very few studies report patient outcomes, which should be an important consideration when researching effectiveness in healthcare. The present review should be considered as a first step toward understanding MHP training in psychological skills, and it is important that future work considers the heterogeneity that existed across studies in the present review. Type and content of training, trainee profession, skills, length, type of patients and patient outcomes were varied throughout, and although this heterogeneity provides a much-needed broad overview, it also limits the direct comparison of study findings. As such, more targeted and controlled studies would provide more nuanced understandings.

### Limitations of the Review

The strength of the conclusions of this review has been severely curtailed by the heterogeneity present within the studies, and they should be treated as tentative first steps towards a more solid evidence base. The lack of a sufficient number of controlled studies has prevented a meta-analysis and thus a more definitive conclusion on what variables form the active ingredient for effective learning. For example, many studies using group training combined this with individual learning activities, but it has not been possible to tease out potential effects. This in itself should be the subject of a much larger publication. The generalisability of the findings is also limited, as it is unknown to what extent the particular type of MHP may influence the effectiveness of a training method, or indeed the specific type of patient.

The wider application of some training methods may be difficult in the average mental health care setting, limiting their value beyond this paper. For example, Amerson (2012) uses an international service-learning project to teach cultural competences, yet it is largely impractical for most services to routinely send trainees to other countries. While this is not unheard of, for example the European Hospital and Healthcare Federation HOPE Exchange Programme has been encouraging similar activities since 1981 (European Hospital and Healthcare Federation, 2013), it is not common. In these cases, as with most training processes, adaptations would need to be made to accommodate individual services practices.

### Conclusion

In sum, this review can be said to provide an important platform for skills training and development among MHPs. The work shows a variety of training methods that may be used. It also points to important avenues for further and future considerations. Overall, it is hoped the this review will serve as an integral first step in the improvement of much needed skills training in the area of mental health, which, ultimately, is intended to benefit the wide range of individuals who engage with mental health services across Europe.

## Appendix: Summary of Findings for Effectively Training Healthcare Staff

The following points are based on research of training methods that have had a positive impact on both patients and staff. However, as patient effects are dependent on the particular staff skill being taught, it is not within the scope of this document to make recommendations for training methods that will improve particular patient outcomes. These suggestions are based on a limited number of heterogeneous studies from a narrative analysis, and should be treated with the appropriate amount of caution.

### Individual Training

Refers to self-paced, independent learning, or training of one individual by another.

**Most effective for:** short-term skill enhancement; greater staff self-efficacy and passion for work; improved patient outcomes from single-session contacts early in care pathway; learning where dedicated facilities and funds are not available.

**Not recommend for:** trainees with low self-motivation; long-term skill change; consistent practice.

1. Training should be combined with individualised feedback where possible, in order to reduce inconsistencies in practice. This feedback should reflect current professional guidelines, e.g. be consistent with patient-centred care.
2. Short-term individual training alone is unlikely to create sustained skill change, but may enhance passion for work-related learning that can be translated into job-based motivation.
3. Trainees should already have a relatively high level of motivation for self-directed learning.

### Group Training

Refers to either formal structured training with other individuals, or on-the-job learning by working within a team. Groups can be interactive, e.g. using role plays, or passive, such as didactic lectures.

**Most effective for:** outcomes that are affected by the availability of professional knowledge, e.g. diagnostic, supervision, communication, consistency in practice, and prescription skills. Creates natural environments for ongoing group learning.

**Not recommend for:** patient outcomes unrelated to breadth of MHP knowledge.

1. Training should include interactive elements, such as role plays, group discussion, and peer presentations. Less structured interactive activities can also be considered, such as peer feedback at the end of a shift.
2. A level of collaborative ability should already be present among group members. If not, consider reducing the amount of interactive work.
3. Where possible, groups should include individuals from a range of related professions. One example of this is a multi-disciplinary team.
4. Clinical-competency training should be conducted over a longer period of time of at least 6 months, in order to increase the potential of sustained learning.
5. Where possible, peer presentations should be given by qualified professionals to members of related disciplines.

### Web-Enabled Video Training

Refers to the use of digital equipment to either: allow synchronous communication between individuals who are not in the same physical space, or record and review staff performance, both for the purpose of learning.

**Most effective for:** facilitating knowledge-based abilities through group interaction, e.g. communication, when training members are not co-located.

**Not recommend for:** instances where face-to-face communication is also available; where the necessary equipment is not readily accessible; one-to-one learning.

1. Where possible, this type of training should follow the same guidelines as Group Training.
2. Consider making allowances for the different styles of communication required by digital media. For example, eye contact is more difficult and facial nuances less apparent, which may impact activities such as role play.

### Effective Delivery of Training Interventions

One of the most common issues with training is translating learning into practice. Insufficient consideration of how to achieve this increases the chance of inconsistent work and misinformation on what constitutes appropriate care. These problems can be reduced using several strategies that apply regardless of the training method used.

1. Contradictions in practice should be minimised where possible. To this end, providing the following to both trainees and educators should be considered:
  - clear guidelines for practice, professional standards, and learning outcomes. These must be institution-specific where appropriate.
  - peer support, preferably in group format, where discussion of disparities and good practice is encouraged.
  - awareness of an individual's own instances of good practice and departures from it through feedback.
  - coordination and communication of work within teams.
  - collaboration between supervisors, trainees and MDTs, where appropriate.
2. Where possible, training should include:
  - individual feedback on work
  - structured learning
  - regular training
3. Where structured training is not available, such as during placement work, suitable supervision should be arranged. Consider having supervisors responsible for:
  - allocating work for trainees that is appropriate to their skill-level, i.e. using supervisors as 'pace-makers'.
  - overseeing trainee work where there is relatively high risk to patients from incorrect procedures.
  - providing regular feedback to trainees.
4. Supervisors must be approachable by trainees, and subject to the same guidance as in point
5. Where particular patient groups are identified as especially challenging to work with, provide additional training and information specific to them.
  - This is particularly effective when targeted at the initial assessment appointment.
  - Inter-disciplinary discussion of these groups should be encouraged.
6. Consider what type of change is desired:
  - long-term patient change can be achieved through short-lived staff outcomes, particularly when this occurs early in the care pathway.
  - consider brief individual training if short-term staff change is required. This can be delivered through relatively cost-effective means, such as handouts.
  - consider group training for more sustained staff change. Where staff are likely to have prolonged contact with patients, such as through therapy, include long follow-up periods of training, such as every six months over a one year period.

## References

[r1] Aiarzaguena J. M. Gaminde I. Grandes G. Salazar A. Alonso I. Sanchez A. (2009). Somatisation in primary care: Experiences of primary care physicians involved in a training program and in a randomised controlled trial. BMC Family Practice*,* 10, . Retrieved from http://www.biomedcentral.com/1471-2296/10/73 19930729 10.1186/1471-2296-10-73 PMC2790434

[r2] Alonso J. Angermeyer M. C. Bernert S. Bruffaerts R. B. Brugha T. S. Bryson H. Vollebergh W. A. M. (2004). Use of mental health services in Europe: Results from the European Study of the Epidemiology of Mental Disorders (ESEMeD) project. Acta Psychiatrica Scandinavica, 109(Suppl. s420), 47–54. 10.1111/j.1600-0047.2004.00325.x 15128387

[r3] American Psychiatric Association. (2013). *Diagnostic and statistical manual of mental disorders* (5th ed.). Washington, DC: Author.

[r4] Amerson R. (2012). The influence of international service-learning on transcultural self-efficacy in baccalaureate nursing graduates and their subsequent practice. International Journal of Teaching and Learning in Higher Education, 24(1), 6–15. Retrieved from http://www.isetl.org/ijtlhe/pdf/IJTLHE1102.pdf

[r5] Bakker I. M. van Marwijk H. W. J. Terluin B. Anema J. R. van Mechelen W. Stalman W. A. B. (2010). Training GP’s to use a minimal intervention for stress-related mental disorders with sick leave (MISS): Effects on performance: Results of the MISS project; a cluster-randomised controlled trial. Patient Education and Counseling, 78, 206–211. 10.1016/j.pec.2009.07.006 19647973

[r6] Bregnhøj L. Thirstrup S. Kristensen M. B. Bjerrum L. Sonne J. (2009). Combined intervention programme reduces inappropriate prescribing in elderly patients exposed to polypharmacy in primary care. European Journal of Clinical Pharmacology, 65, 199–207. 10.1007/s00228-008-0558-7 18807252

[r7] Campbell R. Pound P. Pope C. Britten N. Pill R. Morgan M. Donovan J. (2003). Evaluating meta-ethnography: A synthesis of qualitative research on lay experiences of diabetes and diabetes care. Social Science & Medicine, 56, 671–684. 10.1016/S0277-9536(02)00064-3 12560003

[r8] Challis, S. (2013). *The Mind guide to Who's Who in mental health*. London, United Kingdom: Mind.

[r9] Collin K. M. Valleala U. M. Herranen S. Paloniemi S. (2012). Ways of interprofessional collaboration and learning in emergency work. Studies in Continuing Education, 34(3), 281–300. 10.1080/0158037X.2011.617364

[r10] Critical Appraisal Skill Programme. (2013). *Qualitative Research Checklist* [Critical appraisal tools set]. Retrieved from www.casp-uk.net/#!casp-tools-checklists/c18f8

[r11] de Feijter J. M. de Grave W. S. Dornan T. Koopmans R. P. Scherpbier A. J. J. A. (2011). Students’ perceptions of patient safety during the transition from undergraduate to postgraduate training: An activity theory analysis. Advances in Health Sciences Education: Theory and Practice, 16(3), 347–358. 10.1007/s10459-010-9266-z 21132361 PMC3139877

[r12] de Haes H. Tunissen S. (2005). Communication in palliative care: A review of recent literature. Current Opinion in Oncology, 17(4), 345–350. 10.1097/01.cco.0000167735.26454.79 15933465

[r13] Department of Employment. (1971). *Glossary of training terms.* London, United Kingdom: Her Majesty’s Stationary Office.

[r14] Department of Health. (2011a). No health without mental health: Delivering better mental health outcomes for people of all ages. Retrieved from https://www.gov.uk/government/uploads/system/uploads/attachment_data/file/215811/dh_124057.pdf

[r15] Department of Health. (2011b). No health without mental health: A cross-government mental health outcomes strategy for people of all ages. Retrieved from https://www.gov.uk/government/uploads/system/uploads/attachment_data/file/213760/dh_123990.pdf

[r16] Department of Health. (2013). Delivering high quality, effective, compassionate care: Developing the right people with the right skills and the right values. Retrieved from http://hee.nhs.uk/wp-content/uploads/sites/321/2013/05/29257_2900971_Delivering_Accessible.pdf

[r17] Department of Health. (2014). Closing the gap: Priorities for essential change in mental health. Retrieved from https://www.gov.uk/government/uploads/system/uploads/attachment_data/file/281250/Closing_the_gap_V2_-_17_Feb_2014.pdf

[r18] Engeström, Y. (2006). Activity theory and expansive design. In S. Bagnara & G. Crampton Smith (Eds.), *Theories and practice of interaction design* (pp. 3-23). Mahwah, NJ: Lawrence Erlbaum.

[r19] European Hospital and Healthcare Federation. (2013). *HOPE Exchange Programme* Retrieved from http://www.hope.be/04exchange/exchangefirstpage.html

[r20] European Parliament. (2009). *Motion for a European Parliament Resolution on Mental Health* (A6-0034/2009). Retrieved from http://www.europarl.europa.eu/sides/getDoc.do?pubRef=-//EP//TEXT+REPORT+A6-2009-0034+0+DOC+XML+V0//EN

[r21] Feder G. Davies R. A. Baird K. Dunne D. Eldridge S. Griffiths C. Sharp D. (2011). Identification and Referral to Improve Safety (IRIS) of women experiencing domestic violence with a primary care training and support programme: A cluster randomised controlled trial. Lancet, 378, 1788–1795. 10.1016/S0140-6736(11)61179-3 22000683

[r22] Fossli Jensen B. Gulbrandsen P. Dahl F. A. Krupat E. Frankel R. M. Finset A. (2011). Effectiveness of a short course in clinical communication skills for hospital physicians: Results of a crossover randomized controlled trial. Patient Education and Counseling, 84, 163–169. 10.1016/j.pec.2010.08.028 21050695

[r23] Francis, R. (2013). *Report of the Mid Staffordshire NHS Foundation Trust Public Inquiry*. London, United Kingdom: The Stationary Office.

[r24] Gater R. Jordanova V. Maric N. Alikaj V. Bajs M. Cavic T. Sartorius N. (2005). Pathways to psychiatric care in Eastern Europe. The British Journal of Psychiatry, 186, 529–535. 10.1192/bjp.186.6.529 15928365

[r25] Gongora-Ortega J. Segovia-Bernal Y. Valdivia-Martinez J. J. Galaviz-deAnda J. M. Prado-Aguilar C. A. (2012). Educational interventions to improve the effectiveness in clinical competence of general practitioners: Problem-based versus critical reading-based learning. BMC Medical Education, 12(1), . 22784406 10.1186/1472-6920-12-53 PMC3814588

[r26] Gorecki C. A. Brown J. M. Briggs M. Nixon J. (2010). Evaluation of five search strategies in retrieving qualitative patient-reported electronic data on the impact of pressure ulcers on quality of life. Journal of Advanced Nursing, 66(3), 645–652. 10.1111/j.1365-2648.2009.05192.x 20423399

[r27] Gray S. Plaice C. Hadley S. (2009). Implementing a managed learning environment in the NHS. Records Management Journal, 19(2), 107–116. 10.1108/09565690910972066

[r28] Guldin M.-B. Vedsted P. Jensen A. B. Olesen F. Zachariae R. (2013). Bereavement care in general practice: A cluster-randomized clinical trial. Family Practice, 30(2), 134–141. 10.1093/fampra/cms053 22964078

[r29] Higgins, J. P. T., & Green, S. (2011). *Cochrane handbook for systematic reviews of interventions* [Version 5.1.0]. Oxford, United Kingdom: The Cochrane Collaboration.

[r30] Hinrichs, J. R. (1976). Personnel training. In M. D. Dunnette (Ed.), *Handbook of industrial and organizational psychology* (pp. 861-888). Chicago, IL: Rand McNally.

[r31] Jansink R. Braspenning J. Laurant M. Keizer E. Elwyn G. van der Weijden T. Grol R. (2013). Minimal improvement of nurses’ motivational interviewing skills in routine diabetes care one year after training: A cluster randomized trial. BMC Family Practice, 14, . 10.1186/1471-2296-14-44 23537327 PMC3637576

[r32] The Joanna Briggs Institute. (2014). *The Joanna Briggs Institute Reviewers’ Manual 2014: Methodology for JBI Mixed Methods Systematic Reviews.* Retrieved from http://www.joannabriggs.org/assets/docs/sumari/ReviewersManual_Mixed-Methods-Review-Methods-2014-ch1.pdf

[r33] Karlin B. E. Brown G. K. Trockel M. Cunning D. Zeiss A. M. Taylor C. B. (2012). National dissemination of cognitive behavioral therapy for depression in the Department of Veterans Affairs health care system: Therapist and patient-level outcomes. Journal of Consulting and Clinical Psychology, 80(5), 707–718. 10.1037/a0029328 22823859

[r34] Lester H. Birchwood M. Freemantle N. Michail M. Tait L. (2009). REDIRECT: Cluster randomised controlled trial of GP training in first-episode psychosis. The British Journal of General Practice, 59, e183-e190. 10.3399/bjgp09X420851 19520016 PMC2688067

[r35] Mackey E. Dodd K. (2011). Evaluation and effectiveness of pain recognition and management training for staff working in learning disability services. British Journal of Learning Disabilities, 39, 243–251. 10.1111/j.1468-3156.2010.00661.x

[r36] McLoughlin C. (2010). Concentric circles of containment: A psychodynamic contribution to working in pupil referral units. Journal of Child Psychotherapy, 36(3), 225–239. 10.1080/0075417X.2010.524772

[r37] Monrouxe L. V. Rees C. E. (2012). “It’s just a clash of cultures”: Emotional talk within medical students’ narratives of professionalism dilemmas. Advances in Health Sciences Education: Theory and Practice, 17(5), 671–701. 10.1007/s10459-011-9342-z 22187205

[r38] National Institute for Health and Clinical Excellence. (2011). *Service user experience in adult mental health: Improving the experience of care for people using adult NHS mental health services* (Clinical Guidance Number 136). Retrieved from https://www.nice.org.uk/guidance/cg136/resources/guidance-service-user-experience-in-adult-mental-health-improving-the-experience-of-care-for-people-using-adult-nhs-mental-health-services-pdf

[r39] Nilsen L. L. (2011). Workplace learning among general practitioners and specialists: The use of videoconferencing as a tool. Journal of Workplace Learning, 23(8), 501–517. 10.1108/13665621111174861

[r40] Outcome. (2015). In *Macmillan Dictionary* Retrieved from www.macmillandictionary.com

[r41] Penner L. A. Gaertner S. Dovidio J. F. Hagiwara N. Porcerelli J. Markova T. Albrecht T. L. (2013). A social psychological approach to improving the outcomes of racially discordant medical interactions. Journal of General Internal Medicine, 28(9), 1143–1149. 10.1007/s11606-013-2339-y 23377843 PMC3744315

[r42] Pluye P. Hong Q. N. (2014). Combining the power of stories and the power of numbers: Mixed methods research and mixed studies reviews. Annual Review of Public Health, 35, 29–45. 10.1146/annurev-publhealth-032013-182440 24188053

[r43] Public Health England. (2013). *Our priorities for 2013/14.* Retrieved from https://www.gov.uk/government/uploads/system/uploads/attachment_data/file/192676/Our_priorities_final.pdf

[r44] Ross H. Hardy G. (1999). GP referrals to adult psychological services: A research agenda for promoting needs-led practice through the involvement of mental health clinicians. British Journal of Medical Psychology, 72(1), 75–91. 10.1348/000711299159835 10194574

[r45] Shirazi M. Lonka K. Parikh S. V. Ristner G. Alaeddini F. Sadeghi M. Wahlstrom R. (2013). A tailored educational intervention improves doctor’s performance in managing depression: A randomized controlled trial. Journal of Evaluation in Clinical Practice, 19, 16–24. 10.1111/j.1365-2753.2011.01761.x 21883718

[r46] Simons A. D. Pedesky C. A. Montemarano J. Lewis C. C. Murakami J. Lamb K. Beck A. T. (2010). Training and dissemination of cognitive behavior therapy for depression in adults: A preliminary examination of therapist competence and client outcomes. Journal of Consulting and Clinical Psychology, 78(5), 751–756. 10.1037/a0020569 20873911

[r47] Skill. (2014). In *Cambridge Advanced Learner’s Dictionary.* Retrieved from www.dictionary.cambridge.org

[r48] Suldo S. M. Friedrich A. Michalowski J. (2010). Personal and systems-level factors that limit and facilitate school psychologists’ involvement in school-based mental health services. Psychology in the Schools, 47(4), 354–373.

[r49] Tarn D. M. Paterniti D. A. Orosz D. K. Tseng C. H. Wenger N. S. (2013). Intervention to enhance communication about newly prescribed medications. Annals of Family Medicine, 11(1), 28–36. 10.1370/afm.1417 23319503 PMC3596029

[r50] Thomas J. Harden A. (2008). Methods for the thematic synthesis of qualitative research in systematic reviews. BMC Medical Research Methodology, 8, . 10.1186/1471-2288-8-45 18616818 PMC2478656

[r51] Travado, L., Reis, J. C., Borras, J., & the IPOS-EPAAC Task Force. (2014). *Survey report on the mapping of needs and resources in communication skills and psychosocial care in Europe* (D11). Retrieved from European Partnership for Action Against Cancer website: http://www.epaac.eu/images/END/Final_Deliverables/D11_Survey_report_on_the_mapping_of_needs_and_resources_in_communication_skills_and_psychosocial_care_in_Europe.pdf

[r52] van Weert J. C. M. Jansen J. Spreeuwenberg P. M. M. van Dulmen S. Bensing J. M. (2011). Effects of communication skills training and a question prompt sheet to improve communication with older cancer patients: A randomized controlled trial. Critical Reviews in Oncology/Hematology, 80, 145–159. 10.1016/j.critrevonc.2010.10.010 21075644

[r53] Webb, R. (2011). *Addressing the global health workforce crisis: Challenges for France, Germany, Italy, Spain and the UK* Retrieved from Action for Global Health website: http://www.actionforglobalhealth.eu/fileadmin/AfGH_Intranet/AFGH/Publications/HRH_REPORT_-_SCOPE_WEB_LORES_.pdf

[r54] White E. Winstanley J. (2010). A randomised controlled trial of clinical supervision: Selected findings from a novel Australian attempt to establish the evidence base for causal relationships with quality of care and patient outcomes, as an informed contribution to mental health nursing practice development. Journal of Research in Nursing, 15(2), 151–167. 10.1177/1744987109357816

[r55] Wittchen H. U. Jacobi F. Rhem J. Gustavsson A. Svensson M. Jönsson B. Steinhausen H.-C. (2011). The size and burden of mental disorders and other disorders of the brain in Europe 2010. European Neuropsychopharmacology, 21(9), 655–679. 10.1016/j.euroneuro.2011.07.018 21896369

[r56] World Health Organization, Global Health Workforce Alliance. (2008). *Financing and economic aspects of health workforce scale-up and improvement: Framework paper.* Retrieved from http://www.who.int/workforcealliance/knowledge/resources/rrt_framework/en

